# Supplementary material for: Rationally Designed CdS-Based Ternary Heterojunctions: A Case of 1T-MoS2 in CdS/TiO2 Photocatalyst
Source: Nanomaterials (Basel). 2020 Dec 25;11(1):38. doi: 10.3390/nano11010038 (PMC7823533; doi:10.3390/nano11010038)
Supplement: Supplementary file 1 [file nanomaterials-11-00038-s001.pdf]

nanomaterials

Supplementary Materials

Rationally Designed CdS-Based Ternary Heterojunctions: A Case of 1T-MoS_2 in CdS/TiO_2 Photocatalyst

Wenqian Chen [1,3,*,†], Shaomei Zhang [1,2,†], Ganyu Wang [1,2], Gang Huang [4], Zhichong Yu [1,2], Yirui Li [1,2] and Liang Tang [1,2,*]

1 Key Laboratory of Organic Compound Pollution Control Engineering, Ministry of Education, Shanghai 200444, China
2 School of Environmental and Chemical Engineering, Shanghai University, Shanghai 200444, China; shaomeizhang@shu.edu.cn (S.Z.); wgy@shu.edu.cn (G.W.); zhichongyu@shu.edu.cn (Z.Y.); yiruili@shu.edu.cn (Y.L.)
3 Shanghai Institute of Applied Radiation, Shanghai University, 20 Chengzhong Road, Shanghai 201800, China
4 Physical Science and Engineering Division King Abdullah University of Science and Technology (KAUST), Thuwal 23955-6900, Saudi Arabia; gang.huang@kaust.edu.sa
* Correspondence: wenqianchen@shu.edu.cn (W.C.); tang1liang@shu.edu.cn (L.T.)
† Equal contribution.

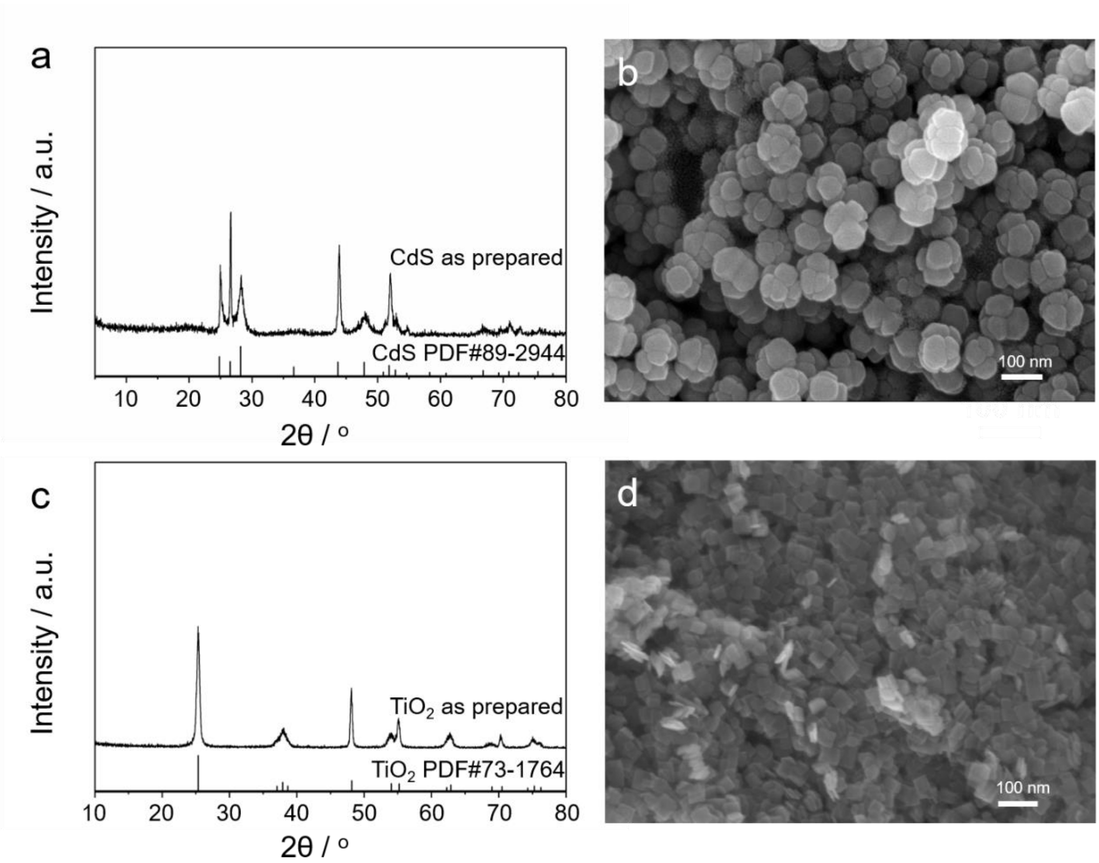

Figure S1. (**a**) XRD pattern and (**b**) SEM image of the pure CdS nanoparticles, (**c**) XRD pattern and (**d**) SEM image of the TiO_2 nanosheets.

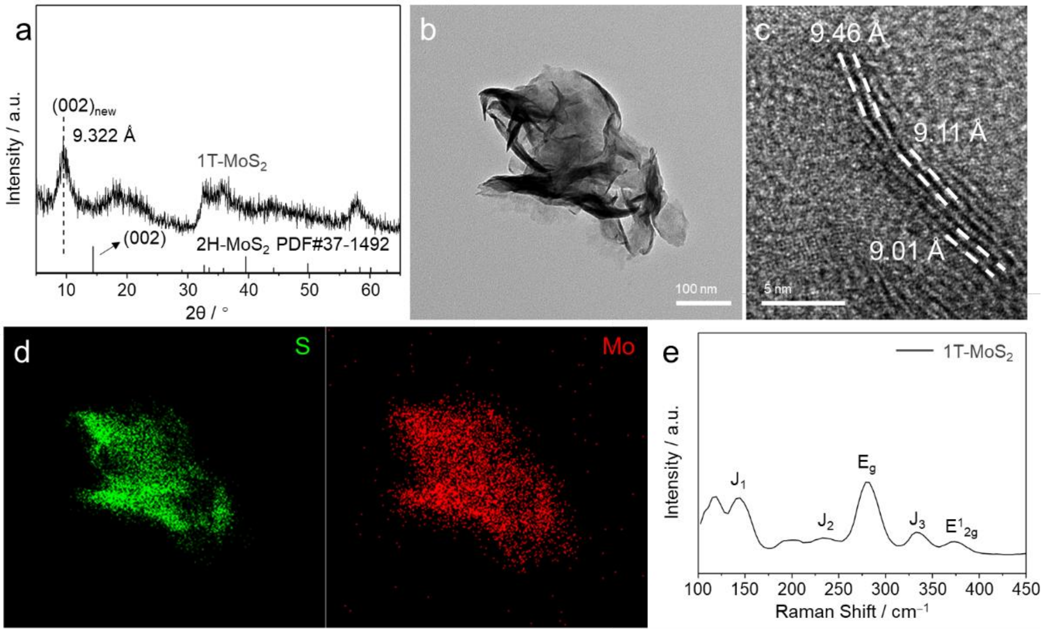

Figure S2. (**a**) XRD pattern, (**b**) TEM image, (**c**) HRTEM image, (**d**) EDS element mapping images of S, Mo, and (**e**) Raman spectra of 1T-MoS_2 nanosheets. Different with 2H phase (JCPDS card No. 37-1492), 002 peak of 1T phase shifted to a low angle of 9.48° (this is corresponding to an interlayer distance of 9.32 Å), and the second-order diffraction peak of 18.31° occurred simultaneously [1]. The d-spacing difference of 002 peak between the 2H and 1T phase is 3.17 Å, which is due to the intercalation of NH_4^+ between MoS_2 layers [2]. HRTEM shown the 1T-MoS_2 nanocrystals were composed of 3–6 layers, with the maximum interval of 9.46 Å, consistent with the XRD results. TEM mapping revealed the uniform distribution of Mo, S elements in the 1T-MoS_2 nanosheet. These results represent the successful synthesis of 1T-MoS_2.

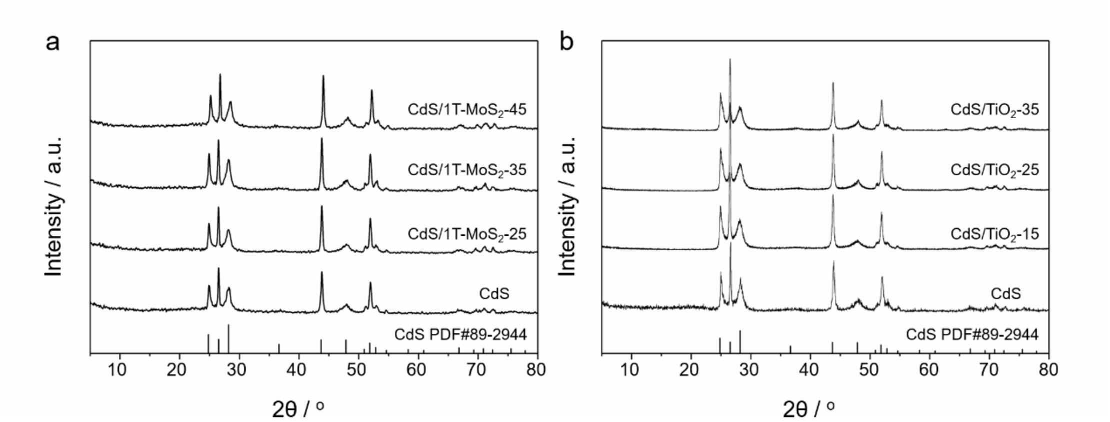

Figure S3. XRD patterns of binary heterojunctions CdS/1T-MoS_2 (**a**) and CdS/TiO_2 (**b**).

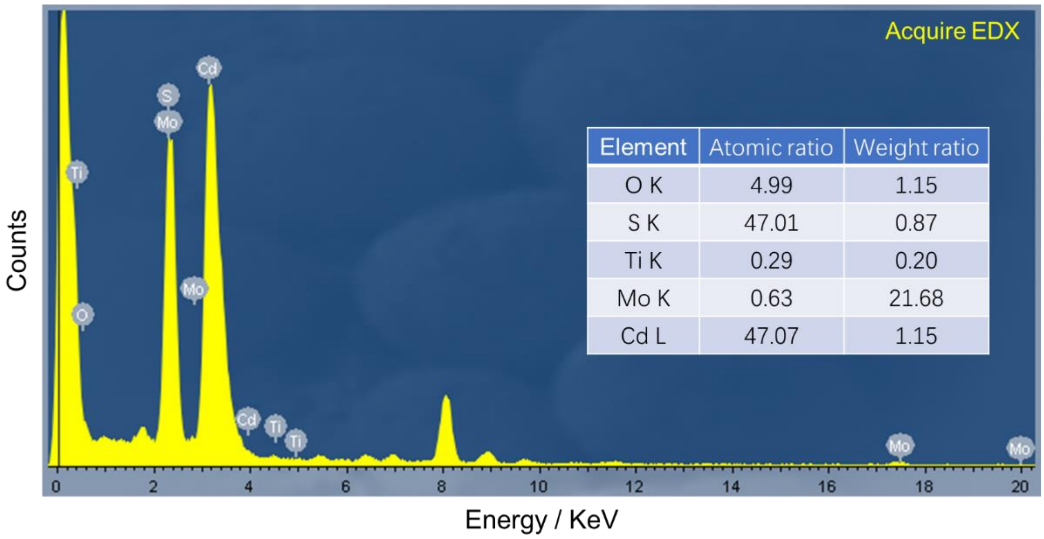

Figure S4. EDS spectrum of CdS/1T-MoS2/TiO2-45-15 heterojunctions.

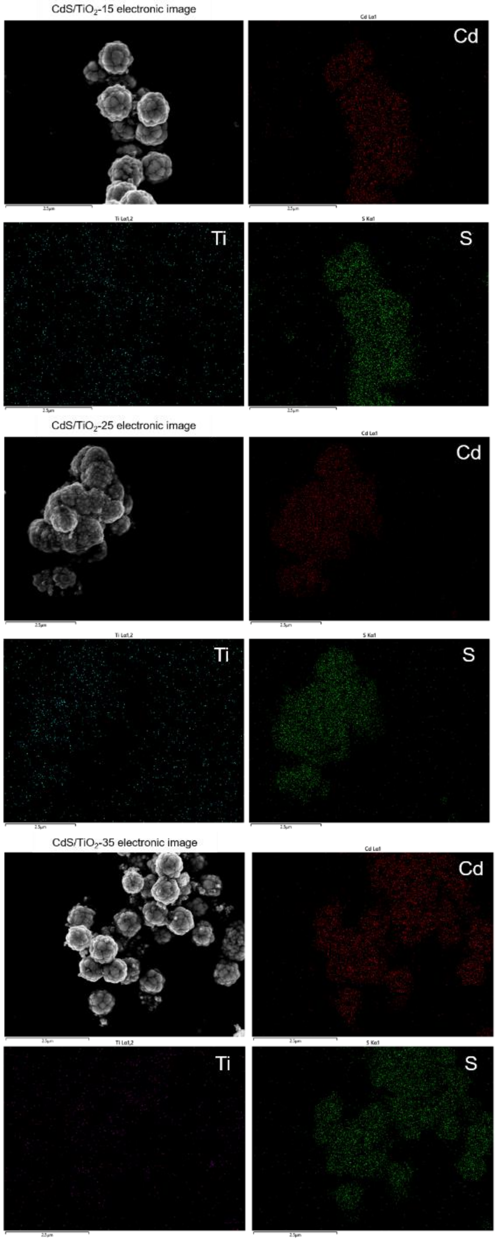

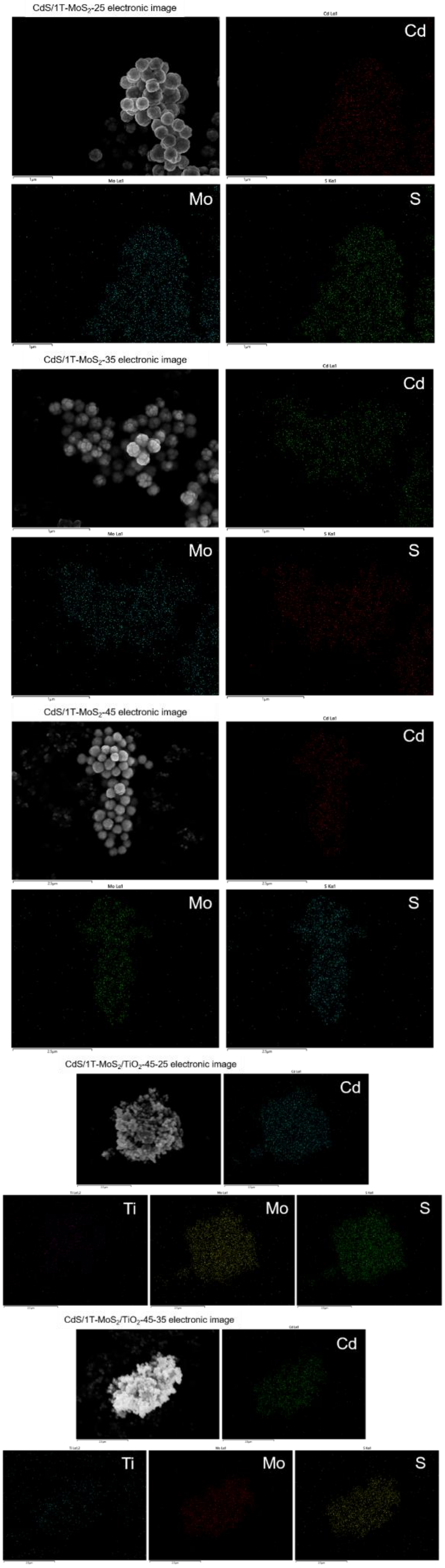
CdS/1T-MoS2-25 electronic image
Cd
Mo
S
CdS/1T-MoS2-35 electronic image
Cd
Mo
S
CdS/1T-MoS2-45 electronic image
Cd
Mo
S
CdS/1T-MoS2/TiO2-45-25 electronic image
Cd
Ti
Mo
S
CdS/1T-MoS2/TiO2-45-35 electronic image
Cd
Ti
Mo
S

Figure S5. SEM-EDS image of CdS/ TiO_2, CdS/1T-MoS_2 and CdS/1T-MoS_2/TiO_2. EDS element mapping shows the existence of Cd, S, Mo, Ti and their uniform distribution in the composites.

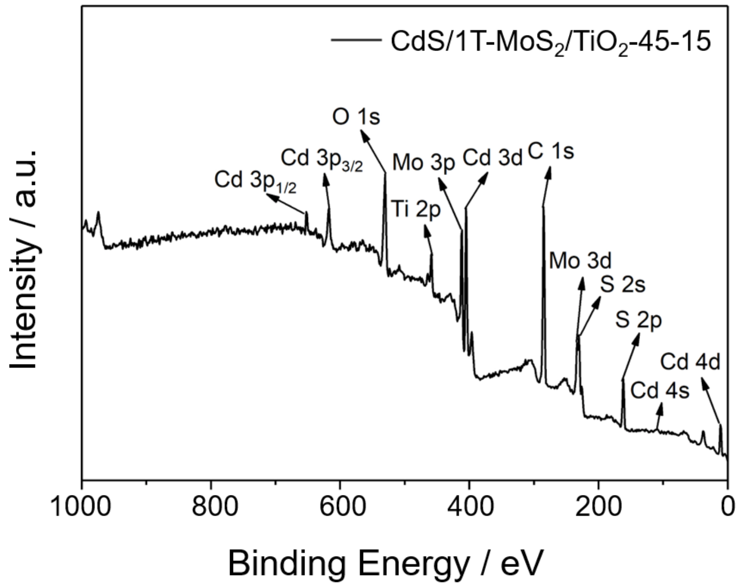

Figure S6. Full XPS spectrum of CdS/1T-MoS_2/TiO_2-45-15.

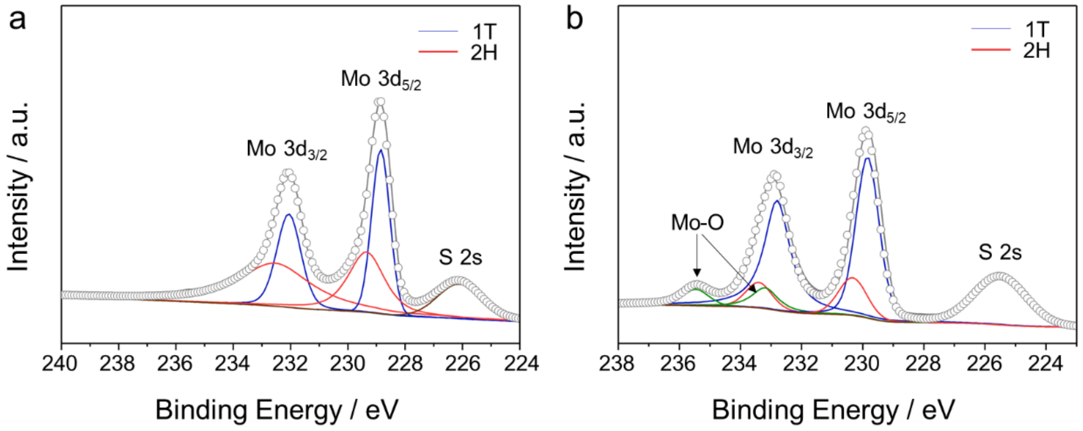

Figure S7. Deconvolutions of Mo 3d XPS analyses of 1T-MoS_2 (**a**) and CdS/1T-MoS_2/TiO_2-45-15 (**b**) samples.

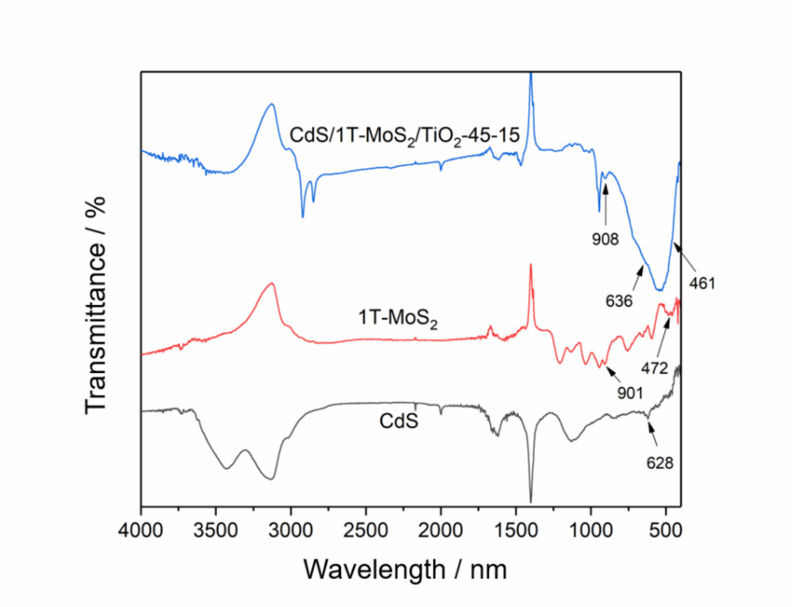

Figure S8. FTIR of pure CdS, 1T-MoS2 and CdS/1T-MoS2/TiO2-45-15 nanocomposites. The IR characteristic peak of CdS at 628 cm^{-1} is attributed to the stretching frequency of the Cd-S bond [3]. The peak around 472 cm^{-1} was associated with Mo-S and the peaks at 901 cm^{-1} were assigned to Mo-O vibrations of 1T-MoS2 [4]. For CdS/1T-MoS2/TiO2-45-15, the corresponding Mo-S and Cd-S peaks were blue shifted. The characteristic peak at 461 cm^{-1} is in accordance with the presence of Ti-O bond [5]. The Raman spectra (Figure 3a) also indicated that there are three the above-mentioned components in our composite.

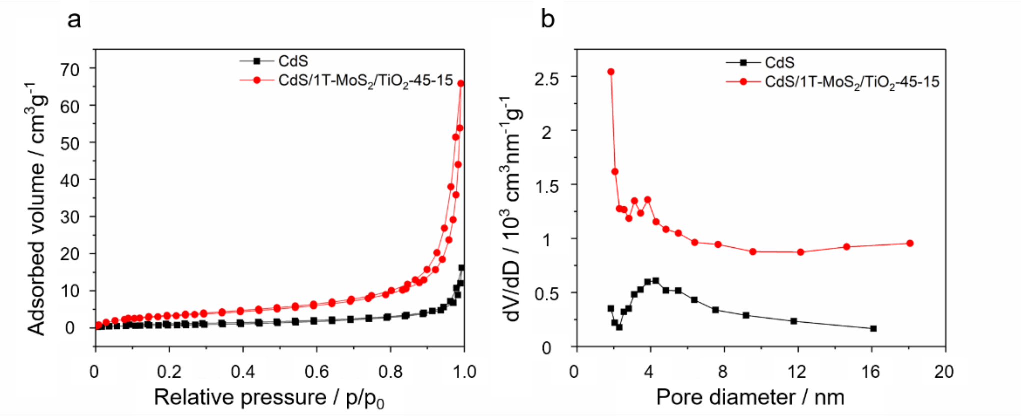

Figure S9. (**a**) Nitrogen sorption isotherms and (**b**) corresponding pore size distribution curves of CdS and CdS/1T-MoS2/TiO2-45-15. N2 adsorption-desorption isotherms of CdS and CdS/1T-MoS2/TiO2 demonstrate a typical type-IV curve, indicating a mesoporous structure of the composite. Brunauer-Emmett-Teller surface area and pore size of CdS nanoparticles are calculated to be ≈2.85 m^2 g^{-1} and 26.78 nm, respectively, which are smaller than that of CdS/1T-MoS2/TiO2 heterojunction (≈12.31 m^2 g^{-1} and 31.43 nm, respectively), revealing the successful loading of 1T-MoS2 and TiO2 on the mesoporous CdS.

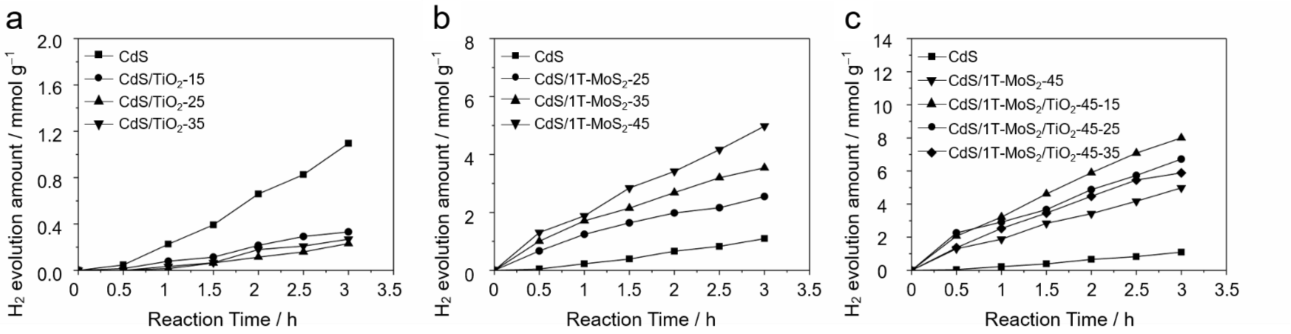

Figure S10. Total amount of photocatalytic hydrogen production in 3h of (**a**) CdS/TiO_2, (**b**) CdS/1T-MoS_2, and (**c**) CdS/1T-MoS_2/TiO_2.

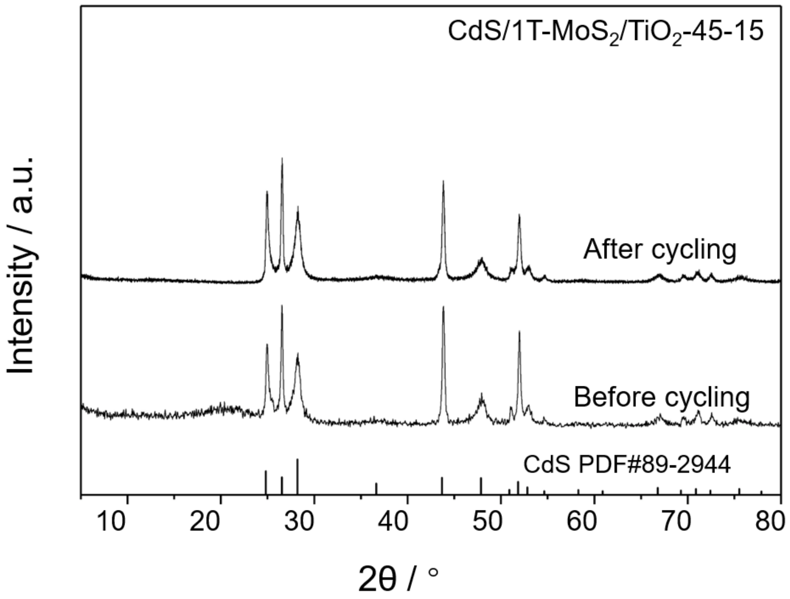

Figure S11. XRD pattern after hydrogen production cycle of CdS/1T-MoS_2/TiO_2-45-15.

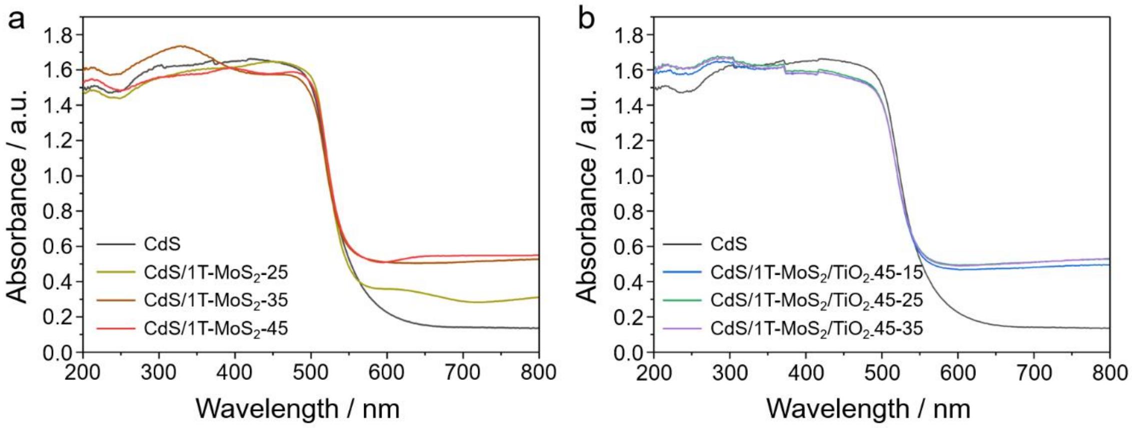

Figure S12. UV-vis diffuse reflectance spectra (DRS) of (**a**) binary CdS/1T-MoS_2 and (**b**) ternary CdS/1T-MoS_2/TiO_2.

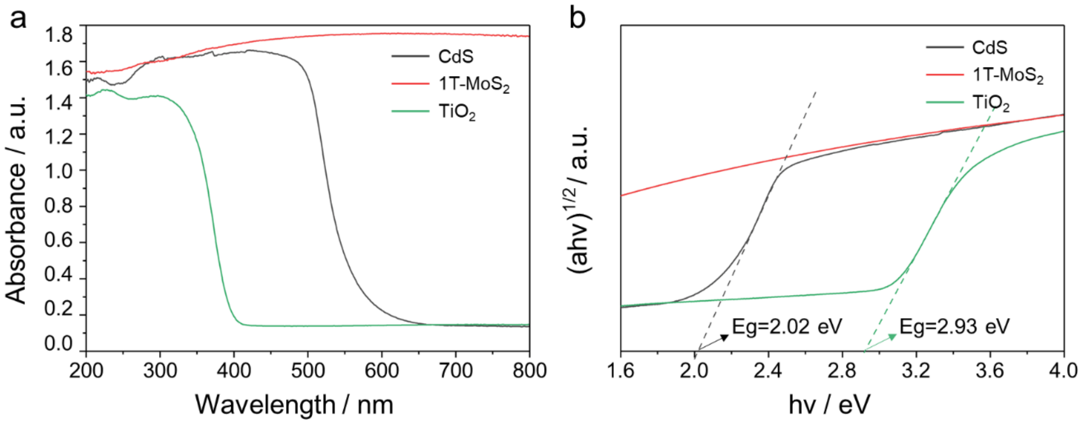

Figure S13. (**a**) UV-vis diffuse reflectance spectra (DRS) and (**b**) The plot of $(\alpha h\nu)^{1/2}$ vs photo energy ($h\nu$) of CdS, 1T-MoS_2 and TiO_2.

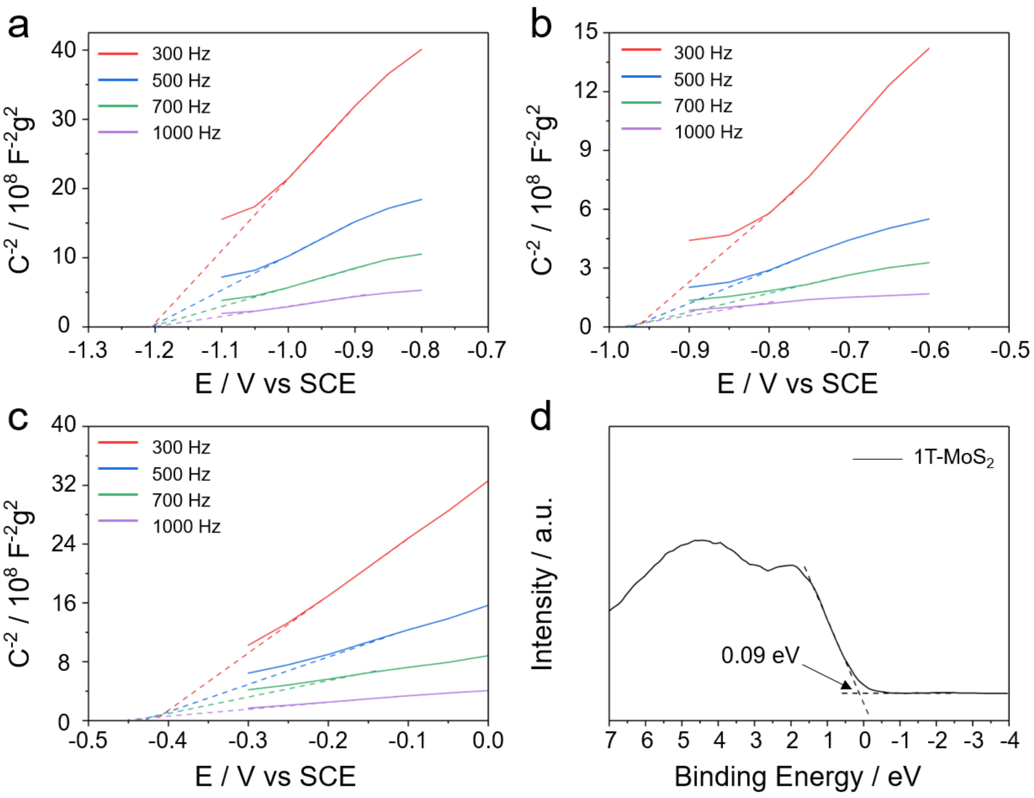

Figure S14. Mott-Schottky plots at different frequencies of (**a**) CdS, (**b**) TiO_2, and (**c**) 1T-MoS_2. (**d**) XPS-VB spectra of 1T-MoS_2.

Reference

1. M.Wu.; J.Zhan.; K.Wu.; Z.Li.; L.Wang.; B.Geng.; L.Wang.; D.Pan.; *J. Mater. Chem. A* **2017**, *5* 14061–14069.
2. A.Anto.Jeffery.; C.Nethravathi.; M.Rajamathi.; *J. Phys. Chem. C* **2014**, *118*, 1386–1396.
3. L. Zhang.; X. Li.; Z. Mu.; J. Miao.; K. Wang.; R. Zhang.; S. Chen.; *RSC Advances* **2018**, *8*, 30747–30754.
4. J. Panda.; B. Tudu.; *AIP Conference Proceedings* **2018**, *1953*, 030127.
5. Mahalingam, T.; Selvakumar, C.; Ranjith Kumar, E.; Venkatachalam, T. *Physics Letters A* **2017**, *381*, 1815–1819.
